# Supplementary material for: Differential Expression of tRNA-Derived Small RNA Markers of Antidepressant Response and Functional Forecast of Duloxetine in MDD Patients
Source: Genes (Basel). 2025 Jan 27;16(2):162. doi: 10.3390/genes16020162 (PMC11855652; doi:10.3390/genes16020162)
Supplement: Supplementary file 1 [file genes-16-00162-s001.zip › genes-3374126-supplementary.pdf]

Supplementary Materials

Differential Expression of tRNA-Derived Small RNA Markers of Antidepressant Response and Functional Forecast of Duloxetine in MDD Patients

Supplementary Table S1. A list of tsRNAs that p-value less than 0.05 when the paired student t-test was evaluated

ts_name	p_values	FC_day8_day0	FDR
tRF.18.HR6HFRD2	0.582577627	1.024867514	0.867426826
tRF.30.PNR8YP9LON4V	0.309686146	0.919579912	0.698578448
tRF.32.PNR8YP9LON4V3	0.077013039	0.810955552	0.390760405
tRF.18.HRERXFD2	0.309825697	0.942202948	0.698578448
tRF.31.PNR8YP9LON4VD	0.001104766	0.75017125	0.052284133
tRF.16.K8KEP1B	0.630250269	0.968767938	0.875201835
tRF.33.PNR8YP9LON4VDP	0.639355219	0.965800033	0.875201835
tRF.17.K879652	0.686016155	0.961927658	0.892084476
tRF.17.DRMD51J	0.289108932	1.039610915	0.698578448
tRF.17.8SOUPR2	0.057940957	1.112228104	0.334763749
tRF.18.YR66IFD2	0.558056914	0.967432507	0.859193648
tRF.16.D9W1X6D	0.433767748	1.044485239	0.786204044
tRF.16.K8E0KRB	0.7174296	0.978317143	0.892084476
tRF.18.476YQSD2	0.302826282	0.933179826	0.698578448
tRF.18.HRE9XFD2	0.213741452	1.091102842	0.624309564
tRF.16.K58Y01B	0.745216365	0.975494026	0.905861809
tRF.33.79MP9P9NH57SD3	0.115967059	1.162895791	0.466164614
tRF.16.DRMD51D	0.535183161	0.972944392	0.854398651
tRF.16.HR6HFRD	0.283962681	1.052140186	0.698578448
tRF.19.VBY9PY11	0.00130311	0.636085606	0.052906261
tRF.17.8SPOX52	0.046001856	0.87040887	0.306176289
tRF.16.KYY8Q1B	0.253790503	1.089810411	0.67788779
tRF.32.79MP9P9NH57SJ	0.407898554	1.062558222	0.775268082

tRF.50.J09PNO2OF5KDYFNP5EYO	0.142204549	1.151170569	0.501768311
tRF.16.K578Q1B	0.313155856	1.086737078	0.698578448
tRF.16.K827K1B	0.502277847	1.049164453	0.82869064
tRF.17.8YQ84V2	0.027115823	0.80353063	0.28633921
tRF.18.H5S8R6D2	0.448571552	0.916345518	0.795033709
tRF.18.299DWUDD	0.920643608	1.007756233	0.96677157
tRF.16.38NJW1B	0.78475467	0.975606923	0.923583208
tRF.17.ON4VN11	0.075117528	0.856322731	0.390760405
tRF.26.SP5830MMUKD	0.825395406	0.985521236	0.940770172
tRF.17.877S6V2	0.159127692	1.154981682	0.52955609
tRF.18.YR6RIFD2	0.071386193	0.884465673	0.381352559
tRF.19.DRMD5112	0.879950012	1.010378987	0.96677157
tRF.16.KVH7K1B	0.392882139	1.067100234	0.766875714
tRF.16.SP5830D	0.41031825	0.937526813	0.775268082
tRF.17.8HWKS52	0.606091364	0.954680873	0.875201835
tRF.20.9LON4VN1	0.000239007	0.535229585	0.020045867
tRF.16.2WMK81B	0.90917659	0.990706405	0.96677157
tRF.16.3KZSP1B	0.671201846	0.955984519	0.890548855
tRF.34.PNR8YP9LON4VHM	0.60171492	0.963533937	0.875201835
tRF.21.73FEWS3VD	0.053217919	1.173032674	0.327370838
tRF.34.79MP9P9NH57S15	0.029187295	1.297349247	0.28633921
tRF.17.8647O52	0.930466382	1.00695366	0.96677157
tRF.16.K8J7K1B	0.713673004	1.024396984	0.892084476
tRF.18.593J24D1	0.404197945	0.943595392	0.775268082
tRF.18.FEWS3VD1	0.924923555	1.009196563	0.96677157
tRF.18.73V6M9DV	0.367720963	1.116032775	0.739082727
tRF.18.8SRZMFD2	0.301310546	0.895938436	0.698578448

tRF.19.DR29NDE2	0.129036118	0.888370039	0.480629942
tRF.31.1OR6QOH04MR1D	0.166171824	1.151787096	0.539726083
tRF.16.K5YY01B	0.185414983	1.114884514	0.572617134
tRF.17.K6MY6V2	0.727527297	0.941476167	0.898400564
tRF.35.PNR8YP9LON4VN1	0.545453005	0.940183208	0.854398651
tRF.31.6978WPRLXN4VE	0.037689143	1.145003166	0.292588588
tRF.16.863IP5D	0.65735773	1.05297992	0.880815969
tRF.22.9P9NH57SJ	0.371328519	1.072838718	0.742657039
tRF.17.0PQ2B52	0.999053312	0.99985089	0.999457145
tRF.17.299DWUI	0.841007669	1.022677862	0.945842421
tRF.18.6978WPDY	0.717924861	0.966917525	0.892084476
tRF.18.S2I7L7DV	0.731421968	0.970085501	0.899870663
tRF.17.86N7O52	0.219863208	1.131955838	0.637603303
tRF.19.WD8YQ8HK	0.130842987	1.107941081	0.482929571
tRF.17.E76INB5	0.044705018	1.185399932	0.302772737
tRF.32.1OR6QOH04MR12	0.143362375	1.210673401	0.501768311
tRF.17.HR6HFRJ	0.454229745	0.940876887	0.795033709
tRF.23.J4S2I7L7DV	0.109433606	0.871145348	0.453367798
tRF.16.K8KQP1B	0.223514905	0.911301857	0.642674493
tRF.16.ML5F92D	0.803559979	1.0211485	0.934800434
tRF.16.4V9K3RB	0.085666645	1.130764356	0.40918421
tRF.20.593J2426	0.58276381	0.961622372	0.867426826
tRF.17.PNR8YPP	0.059463568	0.830714241	0.334763749
tRF.36.PNR8YP9LON4VN1B	0.207412385	0.898838532	0.614667361
tRF.17.PSJR852	0.085021785	1.208639345	0.40918421
tRF.23.79MP9P9NDD	0.360458059	0.916594539	0.734338794
tRF.20.87R8WP9I	0.884196772	1.014587304	0.96677157

tRF.30.87R8WP9N1EWJ	0.194165502	1.106358111	0.588292492
tRF.17.WSNKP92	0.411200176	1.066272686	0.775268082
tRF.18.DRMD51DJ	0.319874992	1.091200814	0.701995929
tRF.19.IRM1DVE2	0.414903758	0.927501732	0.776271547
tRF.16.45LSM1D	0.400289732	1.070849343	0.775268082
tRF.16.48923RB	0.529899221	1.058483335	0.854398651
tRF.29.PNR8YP9LONHK	0.670281786	0.960569275	0.890548855
tRF.19.RPD9W1JV	0.620466478	1.037219845	0.875201835
tRF.17.S2I7L7M	0.902604127	0.988581923	0.96677157
tRF.36.79MP9P9NH57S36D	0.015887294	1.298518746	0.215008048
tRF.17.86J8WPK	0.260294845	0.899380937	0.68468579
tRF.23.9P9NH57SD3	0.080966481	0.861431601	0.400882819
tRF.16.Z92K01B	0.587798025	1.050215485	0.867426826
tRF.17.ZZK0O52	0.044744739	1.430725938	0.302772737
tRF.18.0RER9LD2	0.558540297	1.107357914	0.859193648
tRF.22.18VBY9PYJ	0.264820313	0.912427006	0.68468579
tRF.17.HRERXFJ	0.412457896	0.916337871	0.775268082
tRF.18.H9Q867D2	0.138301283	1.12243575	0.492546674
tRF.18.H9R8O7D2	0.972989948	1.003560655	0.990059947
tRF.19.299DWUFJ	0.611820761	1.048426122	0.875201835
tRF.17.K5KKOV2	0.980946307	0.998110297	0.993177558
tRF.16.K5LYK1B	0.251559038	1.122177113	0.676377281
tRF.16.NMEH62D	0.494747025	0.921477077	0.82869064
tRF.19.593J24FV	0.30096137	0.921744778	0.698578448
tRF.32.6978WPRLXN4VQ	0.881574594	1.012587733	0.96677157
tRF.16.RKIP4OB	0.881001612	1.014260135	0.96677157
tRF.18.RPD9W10M	0.03540448	1.208178175	0.292588588

tRF.20.S2I7L7P5	0.546963327	1.059046022	0.854398651
tRF.24.79MP9P9NF2	0.356963049	0.901130515	0.734338794
tRF.35.79MP9P9NH57S36	0.280376139	1.075793061	0.698578448
tRF.17.8SP6X52	0.326741185	0.907932353	0.706307951
tRF.25.87R8WP9N1E	0.311123846	1.101837091	0.698578448
tRF.17.8US5652	0.04388065	0.810682178	0.302772737
tRF.17.WS7K092	0.958040363	0.995035728	0.98337361
tRF.21.1E6SF8WO0	0.008830233	1.364643492	0.170111817
tRF.50.1OR6QOH04MR1J5KUB4Z3	0.642389524	1.056685276	0.875201835
tRF.26.87R8WP9N1EE	0.638532303	0.949629839	0.875201835
tRF.28.PNR8YP9LOND5	0.106911067	0.850457154	0.452144719
tRF.22.SP5830MMO	0.655765628	0.954962057	0.880815969
tRF.31.79MP9P9NH57SD	0.361577903	1.106130865	0.734338794
tRF.16.K88KO1B	0.036394627	1.595152597	0.292588588
tRF.50.ROD8N0X0JYOYUE4BOPRN	0.013459708	1.230866214	0.193098649
tRF.18.SP5830D4	0.535449594	1.080723896	0.854398651
tRF.30.1OR6QOH04MR1	0.028495823	1.257255162	0.28633921
tRF.16.299DWUB	0.651878875	0.948816714	0.880815969
tRF.16.KPM43RB	0.616136413	1.046178172	0.875201835
tRF.16.308HP1B	0.298993979	0.874490461	0.698578448
tRF.32.Z3R918VBY9PYJ	0.892172106	0.987056203	0.96677157
tRF.21.WB8YYWOVD	0.852350465	1.01497556	0.9533176
tRF.50.PNR8YP9LON4VN1EH6KK8	0.000363369	0.746166255	0.024587986
tRF.17.08Q2B52	0.536879409	1.04554743	0.854398651
tRF.21.WB8689SVD	0.040265815	1.075214714	0.302739273
tRF.50.79MP9P9NH57S362VO0SR	0.454304977	1.045563671	0.795033709
tRF.22.WB8647O52	0.263488741	1.053019419	0.68468579

tRF.18.BS68BFD2	0.553881284	0.960974013	0.859193648
tRF.50.Q1Q89P9L8422YRI7XUK8	0.771600048	0.982432382	0.923583208
tRF.21.WB0Q37Q5D	0.735372199	1.012417959	0.901997319
tRF.50.87R8WP9N1EWJQ72S3HPS	0.640265909	0.950631661	0.875201835
tRF.22.WB8689SV2	0.008879242	1.186921004	0.170111817
tRF.18.HR0VX6D2	0.026146031	1.201572567	0.28633921
tRF.18.H7PU4HD2	0.424265676	1.042695944	0.781415276
tRF.16.3KMB01B	0.168448111	1.11548625	0.542777246
tRF.22.8B8SOUPR2	0.044061196	1.100195293	0.302772737
tRF.22.WB08Q2B52	0.718501536	0.974844782	0.892084476
tRF.16.08Q2B5D	0.361743248	0.932549009	0.734338794
tRF.18.YPSV17DJ	0.697117565	0.971361154	0.892084476
tRF.22.WB86N7O52	0.028344788	1.158797054	0.28633921
tRF.22.WE8S68L52	0.628436737	0.971902651	0.875201835
tRF.50.R9J89O9NF5W8E7OMJHHS	0.635252384	1.035093826	0.875201835
tRF.22.WEKSPM852	0.104103551	1.090918702	0.444905702
tRF.18.YRRHQFD2	0.636436633	0.979534694	0.875201835
tRF.16.K8QJP1B	0.418983098	1.051850783	0.780307972
tRF.50.Q99P9P9NH57S362VO0SR	0.011152824	1.164100096	0.181121857
tRF.18.8R6Q46D2	0.781933197	1.01737257	0.923583208
tRF.22.WB0Q37Q52	0.127332363	1.118446907	0.478675364
tRF.19.DRJRWMI2	0.029621298	1.152811888	0.28633921
tRF.50.779PZBHFEERZYQHQ9M73	0.043709759	1.164800679	0.302772737
tRF.33.86V8WPMN1E8Y0E	0.017467325	1.179598025	0.22161668
tRF.18.WB0Q37DW	0.250723044	1.072236802	0.676377281
tRF.34.10I9BZBZOS4YE2	1.82E-07	1.617784657	7.38E-05
tRF.33.87R8WP9N1EWJDW	0.013514217	0.825481178	0.193098649

tRF.17.HR0VX6J	0.776620683	1.026874032	0.923583208
tRF.50.PSQP4PW3FJIKE7UMKKSW	0.022973769	1.149411744	0.274333829
tRF.22.WE8SPOX52	0.187581475	0.882087706	0.572617134
tRF.43.PNR8YP9LON4VN1EHDX	0.008115093	0.829914403	0.170111817
tRF.18.8R6546D2	0.900278233	1.011632456	0.96677157
tRF.17.8S68L52	0.162589426	1.087583581	0.534435363
tRF.50.P4R8YP9LON4VN1EH6KK8	0.182761447	0.897392794	0.570778058
tRF.22.WB8US5652	0.908969964	0.993595208	0.96677157
tRF.18.HRH7MSD2	0.088349333	1.133046788	0.41646488
tRF.22.8EKSP1852	0.88563428	1.01168954	0.96677157
tRF.22.WD8YQ84V2	0.713385133	1.020109008	0.892084476
tRF.36.18YKISQI45LSM1D	0.111710111	1.113132933	0.458124294
tRF.20.8B8SOUPR	0.302391331	1.046405392	0.698578448
tRF.19.WB8647HU	0.269588907	1.080551118	0.68468579
tRF.21.WB8647O5D	0.388673558	0.96198633	0.765669794
tRF.22.W0K672052	0.258154462	1.056002323	0.68468579
tRF.16.KQ3SW1B	0.076245624	1.136058929	0.390760405
tRF.20.WB8689SV	0.007499601	1.153269847	0.170111817
tRF.31.FN8DYDZDL9X1B	0.00279893	1.553018252	0.094697129
tRF.17.KSPM852	0.570249175	1.039297909	0.867426826
tRF.34.87R8WP9N1EWJI5	0.04310206	0.836280353	0.302772737
tRF.29.34HWH3RXSIHM	0.067919415	1.158023456	0.367670432
tRF.17.08P2F52	0.802166817	1.025129608	0.934800434
tRF.17.KSP1852	0.358420115	1.07297037	0.734338794
tRF.16.MBQ4NKD	0.547151845	1.04897068	0.854398651
tRF.33.P4R8YP9LON4VDP	0.947204653	1.005654553	0.979869639
tRF.21.YOHR6HFRD	0.401602648	1.037880885	0.775268082

tRF.17.BS68BFJ	0.213546855	0.899322267	0.624309564
tRF.18.DRJRWMDV	0.710482795	1.015314317	0.892084476
tRF.17.8689SV2	0.034491091	1.209291385	0.292588588
tRF.17.W3FJIK1	0.447950643	0.927485816	0.795033709
tRF.50.5BF900BY4D84KRIMUF04	0.008270297	1.163989956	0.170111817
tRF.22.WB8Y3DUV2	0.829546112	1.016559478	0.940770172
tRF.37.9LON4VN1EH6KK8N	0.008347403	0.784357507	0.170111817
tRF.19.B1RHODE2	0.175831263	1.099611003	0.553391417
tRF.38.PNR8YP9LON4VN18	0.910092765	0.99208728	0.96677157
tRF.34.PSQP4PW3FJI0E5	0.20034517	1.108984254	0.602519547
tRF.19.WB0Q37IO	0.769281851	1.0203624	0.923583208
tRF.21.WD8YQ84VD	0.101175847	1.104516409	0.436993551
tRF.18.BM32LJDJ	0.972035844	1.001602051	0.990059947
tRF.18.H9R8B7D2	0.999457145	1.000049268	0.999457145
tRF.37.V47P596VW631QJJ	0.447229633	1.062177179	0.795033709
tRF.21.WB86N7O5D	0.355251257	0.95577216	0.734338794
tRF.18.HR07MSD2	0.243906023	1.103840501	0.664602989
tRF.16.I3FJQSD	0.640943191	1.055360594	0.875201835
tRF.19.8B8SOUIJ	0.011148259	1.149648702	0.181121857
tRF.17.863IP52	0.037705422	1.225451077	0.292588588
tRF.17.8871K92	0.82860907	0.969951781	0.940770172
tRF.19.WB08Q20U	0.958149834	0.996347245	0.98337361
tRF.16.BS68BFD	0.614641603	0.969931168	0.875201835
tRF.18.07QSNHD2	0.093495341	1.119822711	0.422988585
tRF.18.MBQ4NKDJ	0.146214865	0.909549049	0.503078264
tRF.34.PSQP4PW3FJIKE5	0.298341832	1.119020371	0.698578448
tRF.21.8B8SOUPRD	0.060191511	1.125265803	0.334763749

tRF.19.WB8US5IU	0.224777778	0.897321159	0.642674493
tRF.17.YRRHQFJ	0.931053408	0.993267773	0.96677157
tRF.50.FBQ622EVUIJVK697BOJ8	0.003830169	1.22304769	0.111272874
tRF.32.P4R8YP9LON4V3	0.056735703	0.795218462	0.334763749
tRF.38.U0IO5BKD09Z51UD2	0.00024687	1.466991507	0.020045867
tRF.20.NB8PLML3	0.009217882	1.215042557	0.170111817
tRF.41.PSQP4PW3FJIKE7UMD	0.267716976	1.09162202	0.68468579
tRF.19.70VR31KD	0.046984708	1.222157194	0.307674054
tRF.43.7673FEWS3V2VR0PSDZ	0.00145716	1.341978307	0.053782438
tRF.50.86J8WPMN1E8Y7Z2R1HPS	0.133273121	1.14201247	0.483115062
tRF.45.BZ0IV25Z2IUIX1Q7O6	0.00457409	1.584384585	0.123805376
tRF.36.D4ZWRNU3KQ9MV1B	5.62E-07	1.697292931	0.000113999
tRF.16.08P2F5D	0.269827544	0.904141125	0.68468579
tRF.17.1EWJQ72	0.411800661	1.054447674	0.775268082
tRF.16.HSRVK7D	0.897869326	1.010473987	0.96677157
tRF.34.I8W47W1R7HFEE2	0.145386891	1.325969346	0.503078264
tRF.16.489B3RB	0.060017883	0.866839734	0.334763749
tRF.22.WE884U1D2	0.704659035	0.958649323	0.892084476
tRF.17.BM32LJJ	0.503791234	0.951433364	0.82869064
tRF.18.BS6PDFD2	0.466235216	1.060827858	0.806874999
tRF.50.18YKISQI45LSM1M3WE8S	0.580281458	0.9569161	0.867426826
tRF.40.2VR008R959KUMKF6	0.285526436	0.921266579	0.698578448
tRF.50.6SXMSL73VL4YMY91PJB7	0.869745347	0.990770283	0.96677157
tRF.50.S2I7L7P5QK11M3WE8SPO	0.44377271	0.923064852	0.795033709
tRF.20.VBY9PYKH	0.123138706	0.836206135	0.471644479
tRF.50.DI2OOIQO4QPRJW4DBQ6Z	0.000107696	1.351465212	0.014574858
tRF.50.00BY4D84KRIMUF04QZ45	0.038195062	1.189682502	0.292588588

tRF.18.HSRVK7D2	0.620175837	0.939786494	0.875201835
tRF.50.22EVUIJVK697BOJ8N981	0.064460983	1.159111155	0.353664309
tRF.19.M91MP3HJ	0.092060321	1.224766432	0.422988585
tRF.31.6SXMSL73VL4YD	0.633529766	1.041670101	0.875201835
tRF.50.RX6NMH490VL8K87SIRMM	0.003836996	1.324139036	0.111272874
tRF.35.S2I7L7P5QK11M3	0.709151781	1.032901224	0.892084476
tRF.36.S2I7L7P5QK11M3E	0.099928419	1.188504432	0.436246648
tRF.35.73V2Y8L981PV6R	0.491142015	1.064585547	0.827401072
tRF.18.8B8SOUDV	0.261715927	1.076726156	0.68468579
tRF.41.8L8NRS9NS334L2H1B	0.163226564	0.845990327	0.534435363
tRF.33.86J8WPMN1E8Y0E	0.000450639	1.301572846	0.026137042
tRF.16.8S68L5D	0.706133632	0.968534074	0.892084476
tRF.17.8Y3DUV2	0.81375832	1.020139258	0.938596245
tRF.20.WB86Q3P9	0.334920748	0.917967529	0.715672757
tRF.17.YPSV17J	0.545978021	0.937843006	0.854398651
tRF.17.D9W1X6K	0.350355767	0.92437421	0.734338794
tRF.29.1EWJQ72S3HIJ	0.445802226	1.070580319	0.795033709
tRF.40.18YKISQI45LSM1M3	0.089242474	1.16003803	0.41646488
tRF.50.ISQI45LSM1M3WE8SSP6D	0.429201002	0.874591889	0.781415276
tRF.16.I7L7P50	0.226989915	1.114309794	0.644460876
tRF.50.596VW631QJ3KYUYRR6RB	0.301277543	0.60875836	0.698578448
tRF.27.739P8WQ0D52	0.050510387	1.928445657	0.320425268
tRF.43.79MP9P9NH57S362VD6	0.010499662	1.256964013	0.181121857
tRF.32.87R8WP9N1EWJM	0.118511858	0.881698981	0.468016737
tRF.40.87R8WP9N1EWJQ72S	0.58967932	0.959678061	0.867426826
tRF.20.WB0Q37Q5	0.925060767	1.008613389	0.96677157
tRF.21.WB08Q2B5D	0.783694887	0.981704336	0.923583208

tRF.17.WS72092	0.902581775	1.011381163	0.96677157
tRF.33.9LON4VN1EH6KDJ	0.289571617	0.887228363	0.698578448
tRF.28.Z6EXEY0VWUD2	0.037298483	1.413958382	0.292588588
tRF.16.K8KDP1B	0.373366961	1.0694212	0.743073461
tRF.17.K8Q2B52	0.049518881	0.570885681	0.319121681
tRF.16.3VLIE1B	0.013792761	1.278803108	0.193098649
tRF.35.PSQP4PW3FJIKE7	0.077959588	1.19935322	0.390760405
tRF.22.R29P4P9LL	0.318663771	0.895195642	0.701995929
tRF.16.S3M830E	0.687352463	1.038757344	0.892084476
tRF.18.S8V0J8DR	0.741340374	0.96881723	0.905861809
tRF.39.87R8WP9N1EWJQ7FV	0.923416251	1.008187512	0.96677157
tRF.42.87R8WP9N1EWJQ72SJ	0.582433046	1.037913892	0.867426826
tRF.22.WB8HWKS52	0.469244506	1.064298387	0.806874999
tRF.21.WB8639SVD	0.051705983	1.168367997	0.322963522
tRF.22.WEPSJR852	0.327058854	0.91513843	0.706307951
tRF.22.WE8RO86J2	0.819407611	1.034635839	0.940770172
tRF.19.WE8SPOJU	0.291234049	1.072594226	0.698578448
tRF.19.W3FJIKE5	0.120621447	1.179573606	0.468976369
tRF.18.XRPM46D2	0.572186096	1.066166003	0.867426826
tRF.50.Y8L981PV6RRNLNK88KO1	0.540323238	0.901899842	0.854398651
tRF.22.ZKXU53K8N	0.114339427	0.875832657	0.464218073
tRF.50.EERZYQHQ9M739P8WQ0D5	0.535721342	0.872409214	0.854398651
tRF.30.FN8DYDZDL9X1	0.924980565	0.992877546	0.96677157
tRF.50.1EWJQ72S3HPSR95933JK	0.67439834	0.942799836	0.89187533
tRF.19.HRMF3RE2	0.058859068	0.833412956	0.334763749
tRF.50.2RBZ0IV25Z2IUIX1Q7O6	0.589278047	0.8424762	0.867426826
tRF.22.JMRPFQRD5	0.558687495	0.948536558	0.859193648

tRF.30.3JVIJMRPFQRD	0.601252791	0.950989159	0.875201835
tRF.40.PNR8YP9LON4VN1EH	0.647562841	1.035334631	0.879299376
tRF.44.PNR8YP9LON4VN1EHIS	0.025765886	0.83276408	0.28633921
tRF.26.RPM830MMUKD	0.500356355	0.932960879	0.82869064
tRF.50.73V2Y8L981PV6RRNLNK8	0.681225065	0.94159873	0.892084476
tRF.50.7P596VW631QJ3KYUYRR6	0.639072713	1.05610149	0.875201835
tRF.30.V47P596VW631	0.074839857	0.837801423	0.390760405
tRF.17.8R6Q46J	0.125539837	1.138882687	0.476347421
tRF.31.87R8WP9N1EWJ0	0.702610634	0.962076648	0.892084476
tRF.22.WBK647R52	0.893259831	0.989867329	0.96677157
tRF.19.WB86892K	0.243858769	1.104853651	0.664602989
tRF.20.WB8US565	0.85008073	1.016252781	0.9533176
tRF.20.WD8YQ84V	0.312142627	1.101889015	0.698578448
tRF.22.WEK8Q2B52	0.809563421	1.023152308	0.938596245
tRF.22.WE8SP6X52	0.753737453	0.959285394	0.913484794
tRF.50.Z3R918VBY9PYKHM26RRN	0.132624607	0.865081394	0.483115062
tRF.50.BU9NZ6V6Z3M8ZLSSXUOL	0.656958963	0.876588022	0.880815969
tRF.24.BZBZOS4YE2	0.984237439	0.996466096	0.994030846
tRF.50.D5BU9NZ6V6Z3M8ZLSSXU	0.812129874	0.968174392	0.938596245
tRF.50.FBVWNEB01XNYH2SBUL6J	0.098827496	1.176841591	0.436130037
tRF.19.1EWJQ7FV	0.340914456	0.922470726	0.724666331
tRF.29.18YKISQI451V	0.237786989	0.895856102	0.661243272
tRF.26.ISQI45LSM1D	0.959152585	1.004192834	0.98337361
tRF.19.2EK8E01J	0.92849336	1.006114558	0.96677157
tRF.21.2EK8E0KRB	0.308916695	1.094216981	0.698578448
tRF.50.2YU04DYJIO3ZU3U0IO5B	0.475948438	1.074546239	0.808514919
tRF.20.JMRPFQRD	0.230972421	0.895272388	0.646722779

tRF.18.KR6946D2	0.665528307	0.951379823	0.888830568
tRF.36.3IRW18V6XO59KND	0.012509037	1.251322633	0.193098649
tRF.30.34HWH3RXSINH	0.766358756	1.032177102	0.923269005
tRF.17.MBQ4NKJ	0.483665527	0.935971228	0.81820085
tRF.20.M91MP34R	0.27151333	1.124701721	0.68468579
tRF.30.P4R8YP9LON4V	0.634554382	0.954714196	0.875201835
tRF.39.PNR8YP9LON4VN1EM	0.581368479	1.053366362	0.867426826
tRF.32.6SXMSL73VL4YK	0.948494503	1.004519552	0.979869639
tRF.32.6978WPRLXN48Q	0.22992808	1.110593934	0.646722779
tRF.50.ROHL7X0OBQJE9UHY0DFK	0.765867905	1.029243436	0.923269005
tRF.23.RXPIN24Y7	0.533463939	1.062062799	0.854398651
tRF.41.V47P596VW631QJ3KE	0.021531794	1.17920296	0.264906315
tRF.17.86V8WPK	0.897754187	0.986490646	0.96677157
tRF.34.86V8WPMN1E8Y2Q	0.343404555	1.085144908	0.726157549
tRF.35.86V8WPMN1E8Y7Z	0.136672062	1.140690183	0.491051835
tRF.18.WKXU53DJ	0.511803252	1.067694216	0.837871453
tRF.23.W631QJ3K05	0.681551295	0.955892366	0.892084476
tRF.41.XENDBP1IUUK7VZ0RB	0.999276152	0.999916616	0.999457145
tRF.50.YKISQI45LSM1M3WE8SSP	0.328965972	0.898791974	0.706667643
tRF.42.YP9LON4VN1EH6KK8N	0.543918026	0.945802103	0.854398651
tRF.18.YR66EFD2	0.690440764	0.96854543	0.892084476
tRF.16.YRRHQFD	0.629198776	1.046482753	0.875201835
tRF.16.Y9PYKHD	0.384269112	1.081779593	0.76104029
tRF.33.Z3M8ZLSSXUOLD2	0.892396459	1.026685171	0.96677157
tRF.18.0RSSX7D2	0.358892025	1.094521135	0.734338794
tRF.18.1EWJQ7D1	0.186934674	1.10912952	0.572617134
tRF.21.1SS2P46ID	0.924934531	0.991394615	0.96677157

tRF.16.HR0VX6D	0.094807786	1.193621183	0.422988585
tRF.25.2IUIX1Q7O6	0.032640485	1.418903432	0.292588588
tRF.16.J3LPWRD	0.321990908	1.096877671	0.702840369
tRF.20.LEKQ3SW1	0.880340577	0.989211523	0.96677157
tRF.50.OE0D58ZZJQJYSWRYVMMV	0.170744824	1.127440718	0.543536838
tRF.30.5Z2IUIX1Q7O6	0.036762768	1.40330455	0.292588588
tRF.43.P4R8YP9LON4VN1EHDX	0.03709772	0.83589898	0.292588588
tRF.42.PNR8YP9LON4VN1EH4	0.109001647	0.875821768	0.453367798
tRF.41.PSQP4PW3FJI0E7UME	0.912611253	0.990514114	0.96677157
tRF.42.PSQP4PW3FJIKE7UMJ	0.504154158	1.072664037	0.82869064
tRF.37.QKF1R3WE8RO86J2	0.205750225	0.838070941	0.614224935
tRF.17.Q72S3HM	0.281099605	1.096105581	0.698578448
tRF.33.62Z6EXEY0VWUD2	0.001159008	1.464102726	0.052284133
tRF.23.RKIP4OI3X	0.822960217	0.979241777	0.940770172
tRF.17.RPFQRD5	0.467532676	0.934001974	0.806874999
tRF.18.S7PVRSD2	0.016834534	1.226553888	0.220478096
tRF.19.76RSJMJJ	0.700142492	1.035408553	0.892084476
tRF.50.79MP9PMNH5IS362VO0SR	0.823666451	0.977741207	0.940770172
tRF.39.79MP9P9NH57S36F8	0.460627464	0.943665765	0.802638413
tRF.50.V2Y8L981PV6RRNLNK88K	0.472197784	0.921013744	0.806874999
tRF.30.86J8WPMN1E8Y	0.709073237	1.034738845	0.892084476
tRF.35.87R8WP9N1EWJQ7	0.72801425	1.044223246	0.898400564
tRF.50.897PVP941QKS3W2VR008	0.784818243	0.970276516	0.923583208
tRF.20.WB8647O5	0.996475574	0.999617285	0.999457145
tRF.22.WB86Q3P92	0.978790419	1.002634194	0.993177558
tRF.21.WB8US565D	0.561072641	0.949310054	0.859605631
tRF.22.W087W4SV2	0.158807898	1.132077576	0.52955609

tRF.27.9N1EWJQ72SJ	0.054597286	0.816429394	0.330843253
tRF.39.ZLBS5EOB3ZY61DE2	0.240688987	0.924427131	0.664602989
tRF.17.D7M4615	0.684465558	0.968454588	0.892084476
tRF.19.1SS2P4IR	0.839444172	0.980852793	0.945842421
tRF.30.JMRPFQRDWXHR	0.801061059	0.974605289	0.934800434
tRF.19.LNK8KEI1	0.082430905	0.875178596	0.403216233
tRF.22.490VL8K8N	0.03112509	1.238797156	0.292588588
tRF.18.593JK4D1	0.121286992	1.169990175	0.468976369
tRF.32.PSQP4PW3FJI01	0.58691547	1.05555939	0.867426826
tRF.50.PSQP4PW3FJI0E7UM8KSW	0.425007374	1.077755411	0.781415276
tRF.30.PW5SVP9N15WV	0.426220947	1.074039285	0.781415276
tRF.33.Q99P9P9NH57SD3	0.157944151	1.13641319	0.52955609
tRF.23.S3M8309NS	0.967059149	0.995835095	0.988982404
tRF.21.VBY9PYKHD	0.453111375	1.0744224	0.795033709
tRF.42.86V8WPMN1E8Y7Z2RH	0.472995689	1.057377335	0.806874999
tRF.22.WB863IP52	0.428486144	1.082326276	0.781415276
tRF.21.WB86Q3P9D	0.500337256	1.05579984	0.82869064
tRF.39.WP9N1EWJQ72S3HIJ	0.695439811	0.956966918	0.892084476
tRF.40.XENDBP1IUUK7VZ0R	0.025470682	1.205322982	0.28633921
tRF.24.9LON4VN1EM	0.743991907	1.030387715	0.905861809
tRF.20.YOHR6HFR	0.363860474	0.933578901	0.734961952
tRF.19.8BWS72E3	0.171361368	0.883689188	0.543536838
tRF.35.897PVP941QKS3W	0.30785359	1.105989811	0.698578448
tRF.32.PSQP4PW3FJIK1	0.83892653	1.027062233	0.945842421
tRF.16.8R6546D	0.390378442	0.909211364	0.765669794
tRF.50.JY7383RPD9W1X6L85J3K	0.11873331	0.876070558	0.468016737
tRF.33.PSQP4PW3FJIKW	0.599643763	1.064676438	0.875201835

tRF.19.YPSV171J	0.794706251	1.026009501	0.932516584
tRF.17.Z90668J	0.154194238	1.158786716	0.526074458
tRF.23.EXEY0VWUD2	0.094578467	1.297707614	0.422988585
tRF.38.43RX6NMH490VL8DJ	0.315996175	0.9071951	0.701062553
tRF.27.596VW631QJJ	0.774155214	1.025298793	0.923583208

Supplementary Table S2. Pathway analysis of ten tsRNAs predicted target mRNAs.

KEGG pathway	p-value	#genes	#miRNAs
ECM-receptor interaction	0	29	5
Fatty acid biosynthesis	2.22E-16	4	1
TGF-beta signaling pathway	3.81E-08	53	4
Thyroid hormone synthesis	1.27E-05	6	3
Hippo signaling pathway	1.98E-05	48	7
Mucin type O-Glycan biosynthesis	0.000319	13	5
Proteoglycans in cancer	0.000887	72	6
Signaling pathways regulating pluripotency of stem cells	0.023219	30	5
Thyroid hormone signaling pathway	0.027208	26	4
Glioma	0.038828	25	4
Glycosphingolipid biosynthesis - lacto and neolacto series	0.127747	5	2
Oocyte meiosis	0.160036	18	3
ErbB signaling pathway	0.185508	28	3
Amphetamine addiction	0.253591	22	3
Pathways in cancer	0.263516	38	2
Axon guidance	0.313261	30	3

Wnt signaling pathway	0.379409	37	2
Fatty acid metabolism	0.384138	8	2
Focal adhesion	0.406215	44	2
FoxO signaling pathway	0.416118	32	2
Melanoma	0.454571	14	2
Phosphatidylinositol signaling system	0.497625	15	3
PI3K-Akt signaling pathway	0.513631	32	2
Regulation of actin cytoskeleton	0.539993	19	1
Prostate cancer	0.600077	16	2
Colorectal cancer	0.636356	9	1
Prolactin signaling pathway	0.655039	19	2
Non-small cell lung cancer	0.685269	9	1
mTOR signaling pathway	0.699632	11	1
AMPK signaling pathway	0.701446	21	2
Long-term depression	0.756034	18	2
Adrenergic signaling in cardiomyocytes	0.786198	23	2
Lysine degradation	0.793068	10	1
Pancreatic cancer	0.84629	11	2
Rap1 signaling pathway	0.854757	11	1
Glycosaminoglycan biosynthesis - chondroitin sulfate / dermatan sulfate	0.865502	5	3
Cytokine-cytokine receptor interaction	0.872077	17	2
Long-term potentiation	0.87225	21	2
cAMP signaling pathway	0.939162	27	1
Arrhythmogenic right ventricular cardiomyopathy (ARVC)	0.940339	14	2
Amoebiasis	0.945137	12	2
Glycosaminoglycan biosynthesis - heparan sulfate / heparin	0.956268	3	2
Ubiquitin mediated proteolysis	0.957748	27	2

Insulin signaling pathway	0.960103	17	1
Estrogen signaling pathway	0.965059	2	1
Dopaminergic synapse	0.971934	20	1
Other glycan degradation	0.973182	2	1
cGMP-PKG signaling pathway	0.975565	37	2
Thyroid cancer	0.980744	5	1
Progesterone-mediated oocyte maturation	0.980984	15	1
Chronic myeloid leukemia	0.983008	3	1
Glutamatergic synapse	0.987402	16	1
N-Glycan biosynthesis	0.987657	2	1
Type II diabetes mellitus	0.991998	1	1
Sphingolipid signaling pathway	0.993207	9	1
Viral carcinogenesis	0.994802	15	1
Gap junction	0.995701	17	2
Endocrine and other factor-regulated calcium reabsorption	0.996689	9	1
Biosynthesis of unsaturated fatty acids	0.998274	1	1
MicroRNAs in cancer	0.998564	8	1
Morphine addiction	0.999175	9	1
Platelet activation	0.999413	14	1
p53 signaling pathway	0.999582	13	1
Circadian entrainment	0.999729	17	1
Nicotine addiction	0.999874	7	1
Cocaine addiction	0.999904	6	1
B cell receptor signaling pathway	0.99993	13	1
HTLV-I infection	0.999974	11	1
Glycosaminoglycan biosynthesis - keratan sulfate	0.999997	3	1
Amyotrophic lateral sclerosis (ALS)	0.999997	10	1

Jak-STAT signaling pathway	0.999997	9	1
Metabolism of xenobiotics by cytochrome P450	0.999998	1	1
Alcoholism	0.999999	5	1
Serotonergic synapse	1	14	1
Steroid biosynthesis	1	2	1
Primary bile acid biosynthesis	1	1	1
beta-Alanine metabolism	1	2	1
Glycosaminoglycan degradation	1	1	1
Thiamine metabolism	1	1	1
Pantothenate and CoA biosynthesis	1	1	1
2-Oxocarboxylic acid metabolism	1	2	1
